# Venolymphatic malformation in lateral edge of the tongue: case report

**DOI:** 10.1590/1677-5449.200113

**Published:** 2022-03-07

**Authors:** Erasmo Freitas de Souza, Dáurea Adília Cóbe Sena, Virgínia Raquel dos Santos Lucena, Lélia Batista de Souza, Hécio Henrique Araújo de Morais

**Affiliations:** 1 Universidade do Estado do Rio Grande do Norte – UERN, Programa de Pós-graduação em Saúde e Sociedade, Mossoró, RN, Brasil.; 2 Universidade Federal do Rio Grande do Norte – UFRN, Programa de Pós-graduação em Patologia Oral, Natal, RN, Brasil.; 3 Universidade Federal do Rio Grande do Norte – UFRN, Escola Multicampi de Ciências Médicas, Curso de Medicina, Caicó, RN, Brasil.

**Keywords:** tongue, vascular diseases, vascular malformations

## Abstract

Vascular malformations are vascular anomalies that can affect veins, lymphatic vessels, and/or arteries in isolated or mixed form. When they present in the mixed form with venous and lymphatic involvement, they are called venolymphatic or lymphatic-venous malformations, depending on their predominant component. Although these are benign disorders with good prognosis, they are locally invasive and may lead to deformity, while there is also a propensity for local recurrence. This article presents a case of venolymphatic malformation with unusual localization on the lateral border of the tongue, addressing the clinical conduct and the current theoretical framework.

## INTRODUCTION

Vascular anomalies can be subdivided into vascular tumors or vascular malformations (VM).^1^^-^ 3 Vascular malformations can be further subdivided according to the International Society for the Study of Vascular Anomalies (ISSVA) classification (Table 1).^1^

**Table 1:** Classification of vascular anomalies.

VASCULAR TUMORS	VASCULAR MALFORMATIONS
Benign	Simple
Locally aggressive or borderline	Combined
Malignant	Named according to a major vessel*
	Associated with other anomalies

* Also known as “channel type” or “truncal” vascular malformations.

With regard to VM, these are enduring lesions that grow with the affected individual and are the result of progressively growing abnormal vessel morphology, composed of atypical vascular architecture, such as veins, lymph vessels, arteries, or mixed presentations.^1^^-^ 10

Vascular anomalies are differentiated on the basis of their hemodynamic characteristics as low flow (venous and lymphatic components) or high flow (arterial components). Low flow lesions are soft and compressible on palpation, whereas high flow lesions are firm and may present thrill or murmur, which are characteristics that are only present in lesions of this type, and this differentiation is important for deciding on treatment.^2^^,^ 7 ^-^ 9

When these lesions involve a mixture of venous and lymphatic components, they are called venolymphatic or lymphatic-venous malformations, depending on their predominant component. They are rare anomalies, with etiology that has not been fully elucidated, and generally grow slowly, painlessly, and progressively. While mixed VM are histologically benign and have good prognosis, locally they can invade muscle, bone, and other neighboring tissues, which can cause severe deformity, and there is also a possibility of local recurrence.^1^^-^ 6 ^,^ 8 ^-^ 13

Treatment methods include pharmacological approaches, with steroids and beta blockers, aiming to inhibit angiogenesis and induce capillary regression, sclerosant therapies, electrocoagulation, cryosurgery, laser treatment, embolization, and surgical removal, the last of which is the first choice in the majority of cases.^2^^-^ 7 ^,^ 9 ^,^ 11 ^-^ 15

This study was approved by the Research Ethics Committee at the Universidade do Estado do Rio Grande do Norte (consolidated opinion number: 5.157.375).

## CASE DESCRIPTION

**Figure 1:**
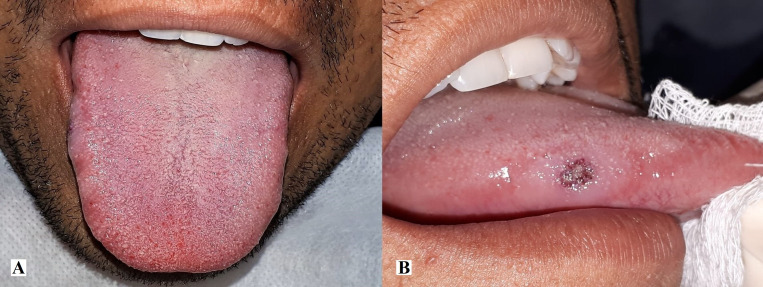
Clinical appearance of the lesion. (A) View of the dorsum of the tongue, showing changed volume and swollen surface of the right lateral border; (B) Right lateral view of the tongue, showing area of ulceration in the center of the lesion.

The patient was a 36-year-old, brown-skinned male, who presented with an abnormal swelling of the right lateral edge of the tongue (Figure 1A), with non-pulsatile consistency, hardened margins, an area of ulceration in the center (Figure 1B), and apparently normal surrounding mucosa. The patient stated that the lesion had been present for more than 2 years, denied using tobacco, alcohol, or other drugs, and reported that he had not suffered any local trauma that could be related to the lesion. His personal and family medical histories did not contribute to diagnosis. The initial diagnostic hypothesis was squamous cell carcinoma, in view of the lesion’s characteristics, including hardened margin and focal ulceration, and its anatomic site; although the 2 years since onset would contradict this hypothesis.

**Figure 2:**
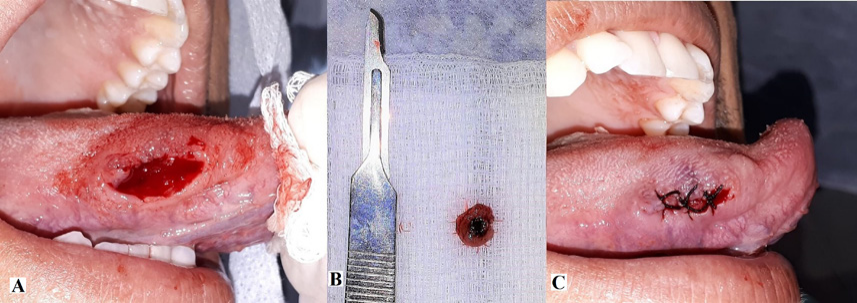
Excisional biopsy of the lesion. (A) Surgical wound after removal of the lesion; (B) Lesion removed; (C) Site sutured.

An excisional biopsy was performed under local anesthesia and in appropriate conditions (Figure 2A). The specimen removed measured 0.3 x 0.3 x 0.3 cm (Figure 2B) and was duly stored in 10% formol and sent for histopathological analysis. The tongue was sutured without complications (Figure 2C) and the patient was maintained under periodic observation. The lesion did not relapse.

**Figure 3:**
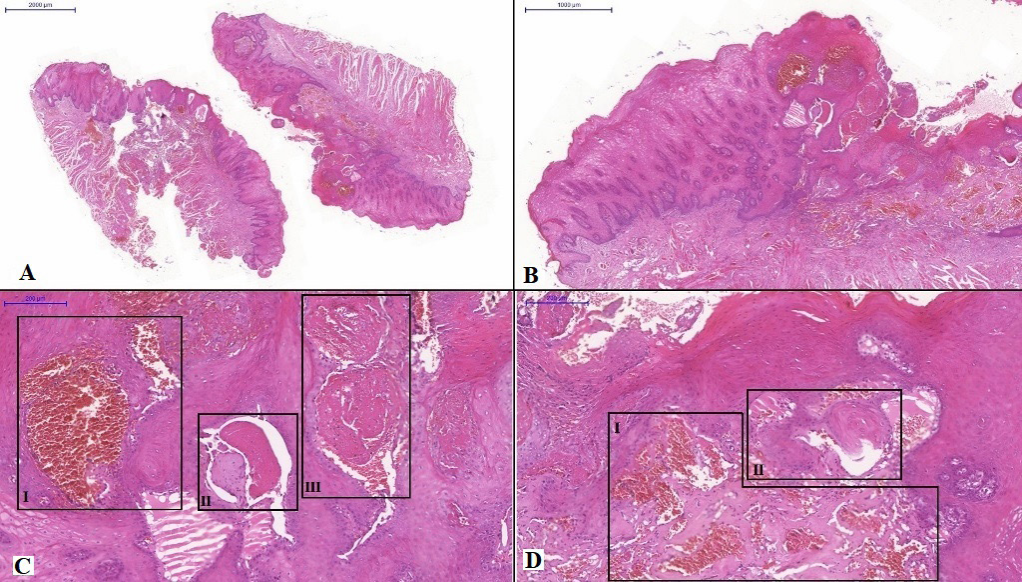
Microphotographs of the lesion. (A) 2000 μm; and (B) 1000 μm. Histological sections stained with hematoxylin and eosin revealed fragments of oral mucosa lined with parakeratinized stratified squamous epithelium with extensive areas of acanthosis, hyperplasia, and hydropic degeneration; CI) 200 μm. Blood vessels of varying sizes, some with congested appearance; CII) 200 μm. Lymph vessels with eosinophilic material suggestive of lymph in their lumina; CIII) 200 μm. Large, dilated, tortuous lymph vessels; DI) 200 μm. Blood vessels of varying sizes, some with congested appearance; DII) 200 μm. Large, dilated, tortuous lymph vessels.

Histological sections stained with hematoxylin and eosin revealed fragments of oral mucosa lined with parakeratinized stratified squamous epithelium with extensive areas of acanthosis, hyperplasia, and hydropic degeneration (Figures 3A and 3B). Large, dilated, tortuous lymph vessels were observed in the subepithelial region (Figures 3C III and 3D II). Some of these vessels had eosinophilic material suggestive of lymph in their lumina (Figure 3C II), with blood vessels of varying sizes, some of which were congested, with a predominance of venule vessels (Figures 3C I and 3D I) and, in some cases, there was dissociation of striated skeletal muscle tissue fibers present in the tissue fragment. The histopathological picture was completed by presence of adipose tissue and striated muscle tissue. The histopathological diagnosis was venolymphatic malformation.

## DISCUSSION

The pathophysiology of VMs is still imprecise and their occurrence is rare. They are present from birth, although they may not be apparent and can remain quiescent throughout adulthood.^6^

In a study with 441 patients with lymphatic malformations, it was observed that 234 were women (53.1%) and 207 were men (46.9%), lesions had been detected at a mean age of 1.9 years, ranging from 0 to 28 years, and the head and neck region was the most often involved in lymphatic malformations (61.2%, n = 270), followed by extremities (17.5%, n = 77), trunk (16.1%, n = 71), and multiple sites (5.2%, n = 23).^16^

In another study, with 614 patients, which analyzed venous malformations, it was observed that 374 were female (60.9%) and 240 were male (39.1%). Lesions had been detected at a mean age of 3.1 years, ranging from 0 to 46 years. The venous malformations involved the extremities (50.3%, n = 309), head and neck (32.7%, n = 201), trunk (9.8%, n = 60), or multiple sites (7.2%, n = 44).^17^

With regard to mixed VM types, these are rarer, particularly so in the stomatognathic system, and only 11 cases have been reported to date, two in the mandible body,^10^^,^ 18 one in a left parotid,^19^ one in the gingiva,^11^ three in left oral mucosa,^3^^,^ 6 ^,^ 7 one in a sublingual position,^20^ and three involving the tongue.^5^^,^ 13 ^,^ 21

The reports listed above involved a wide variation in sex, age, time since onset, and site. Clinical findings in common among these reports, and also with the case described here, include expanding lesions with irregular surfaces and color changes. In the majority of these cases the initial clinical diagnoses were vascular tumors, but there were also suspected carcinomas (as in the present case report), telangiectatic granuloma, and cysts. In common with the case described here, management consisted of surgical removal in all of the cases listed in the preceding paragraph and there was no mention of relapse. However, it is worth pointing out that there is a possibility that multiple treatments may be needed for lymphatic, venolymphatic, or lymphatic-venous malformations because of recurrence.

Mixed malformations in which both lymphatic and blood vessels are involved are generally diagnosed at birth or during the first 2 years of life. There is an increased risk of development in preterms, although there is also evidence of cases with adult presentation that were apparently secondary to trauma.^9^^,^ 12 ^,^ 14

One peculiarity of the present case is that although it was only detected in adulthood, the patient denied having suffered a trauma. However, in addition to the site being prone to trauma, it is also unlikely that a VM that had hitherto been quiescent would initiate a behavioral change with no occurrence of injury, infection, local hemorrhage, or systemic hormonal changes, and the absence of trauma in the patient’s history can be attributed to a lapse of memory linked to the time elapsed between onset of the lesion and his seeking medical care.

With regard to site of presentation, the most common location is the neck, but VM have also been reported in the duodenum, oral cavity and maxillofacial region, colon, bladder, testicle, and spinal column.^9^ Nonetheless, it is rare for a lesion of this size to appear in the oral cavity, particularly in the tongue, and, of the three cases of VM found in the literature, one is a case of lymphatic-venous malformation^13^ and another is subject to retraction because of duplicate publication.^5^ It is therefore important to document clinical cases such as this one, because there is a possibility of confusion when selecting a diagnostic hypothesis, since this is such an uncommon finding.

Clinically, presentation can range from an anomaly that grows slowly over a number of years to an aggressively growing tumor, but without malignant characteristics. The size of these tumors varies because of the different anatomic sites and the relationship to neighboring tissues. The most common complications are random or traumatic hemorrhage, rupture, and infection.^15^

The reasons why VM continue to grow vary. Examples include local trauma, thrombosis, partial resection, and hormonal stimuli, and it is believed that these lesions increase in size by hypertrophy, rather than hyperplastic proliferation. Clinically, it can be difficult to predict how much they will grow, but some lesions appear to expand invasively into surrounding tissues and can even develop into multifocal lesions.^8^

With regard to treatment, although VM may appear similar to hemangiomas, their therapeutic courses differ.^6^ Strategies for diagnosis and treatment of VM should also be based on their flow characteristics, and VM can be subdivided into those with slow or rapid flow, based on the velocity of fluid flow through their capillary systems. Those with venous and lymphatic presentations are considered slow-flowing malformations, while those with arterio-venous presentation are fast-flowing.^8^^,^ 14 ^,^ 21 The present case involved a slow-flowing lesion.

It is recommended that preoperative imaging exams be ordered, including angiotomography, ultrasonography, and magnetic resonance angiography, in order to contribute to diagnosis and planning of the surgical strategy.^14^^,^ 15 ^,^ 21 Surgical removal is the treatment considered most effective; although excision with a safety margin is necessary to prevent recurrence.

## CONCLUSIONS

The complexity of the pathophysiology of VM means that the professional responsible must have the knowledge necessary for adequate diagnosis and therapeutic management. Common clinical findings are expansive lesions with irregular surfaces, color changes, possible associations with trauma, and slow growth, although with a possibility of invading surrounding spaces. Venolymphatic malformation is rarely observed in the tongue, but was diagnosed in the case presented here. The treatment of choice in this case was surgical removal, which is the approach taken in the majority of cases reported in the literature, with no relapses.
