# Supplementary material for: Venolymphatic malformation in lateral edge of the tongue: case report
Source: J Vasc Bras. 2022 Mar 7;21:e20200113. doi: 10.1590/1677-5449.200113 (PMC8958432; doi:10.1590/1677-5449.200113)
Supplement: Translation

# Translation: Malformação venolinfática em borda lateral de língua: relato de caso

**Authors:** Erasmo Freitas de Souza, Dáurea Adília Cóbe Sena, Virgínia Raquel dos Santos Lucena, Lélia Batista de Souza, Hécio Henrique Araújo de Morais

**Affiliations:** 1 Universidade do Estado do Rio Grande do Norte – UERN, Programa de Pós-graduação em Saúde e Sociedade, Mossoró, RN, Brasil.; 2 Universidade Federal do Rio Grande do Norte – UFRN, Programa de Pós-graduação em Patologia Oral, Natal, RN, Brasil.; 3 Universidade Federal do Rio Grande do Norte – UFRN, Escola Multicampi de Ciências Médicas, Curso de Medicina, Caicó, RN, Brasil.

**Keywords:** língua, doenças vasculares, malformações vasculares

## Abstract

As malformações vasculares são anomalias que podem acometer veias, vasos linfáticos e artérias de forma isolada ou mista. Quando se apresentam de forma mista, com componentes venosos e linfáticos, são denominadas malformação venolinfática ou linfático-venosa, de acordo com sua constituição predominante. Embora seja um distúrbio benigno de bom prognóstico, é localmente invasivo, podendo levar a deformidade e havendo, ainda, a propensão de recorrência local. O presente artigo traz um caso de malformação venolinfática com localização incomum em borda lateral de língua, abordando-se a conduta clínica e o referencial teórico vigente.

## INTRODUÇÃO

As anomalias vasculares são lesões que podem ser divididas em tumores vasculares e malformações vasculares (MV)^1^^-^ 3. As MV recebem, ainda, outras subclassificações de acordo com a *International Society for the Study of Vascular Anomalies* – ISSVA (Tabela 1)^1^.

**Tabela 1:** Classificação das anomalias vasculares.

TUMORES VASCULARES	MALFORMAÇÕES VASCULARES
Benignos	Simples
Localmente agressivos ou limítrofes	Combinadas
Malignos	Nomeadas de acordo com o vaso principal*
	Associadas com outras anomalias

* Também conhecidas como “tipo de canal” ou malformações vasculares “tronculares”.

No que concerne as MV, elas são lesões persistentes, que crescem com o indivíduo, resultantes da morfologia anormal dos vasos progressivamente aumentados e compostos de uma arquitetura vascular atípica, como veias, vasos linfáticos, artérias ou com apresentação mista^1^^-^ 10.

As MV são distintas com base nas suas características hemodinâmicas de baixo fluxo (componentes venosos e linfáticos) ou de alto fluxo (componentes arteriais). As lesões de baixo fluxo são moles e compressíveis à palpação, enquanto as de alto fluxo são firmes, além de poderem apresentar frêmito ou sopro, características presentes apenas em lesões dessa natureza, sendo importante essa diferenciação no tocante à terapêutica de eleição^2^^,^ 7 ^-^ 9.

Quando se apresentam de forma mista com componentes venosos e linfáticos, são denominadas malformações venolinfáticas ou linfático-venosas, de acordo com sua constituição predominante. É uma anomalia de desenvolvimento rara, com etiologia controversa, crescimento geralmente lento, indolor e progressivo. Embora as MV mistas sejam histologicamente um distúrbio benigno de bom prognóstico, é localmente invasivo em músculo, osso e demais tecidos subjacentes, podendo levar a grave deformidade, havendo, ainda, a possibilidade de recorrência local^1^^-^ 6 ^,^ 8 ^-^ 13.

As formas de tratamento englobam as modalidades farmacológicas, como o uso de esteroide e betabloqueador, no intuito de inibir a angiogênese e induzir regressão capilar, terapia esclerosante, eletrocoagulação, criocirurgia, laserterapia, embolização e remoção cirúrgica, sendo esta última a conduta de escolha para a maioria dos casos^2^^-^ 7 ^,^ 9 ^,^ 11 ^-^ 15.

O estudo foi aprovado pelo Comitê de Ética e Pesquisa da Universidade do Estado do Rio Grande do Norte (número do parecer consubstanciado: 5.157.375).

## DESCRIÇÃO DO CASO

Paciente do sexo masculino, com 36 anos, feoderma, apresentando alteração de volume de superfície abaulada em borda lateral direita de língua (Figura 1A), de consistência não pulsátil, margens endurecidas e área ulcerada ao centro (Figura 1B), com mucosa circundante apresentando aspecto de normalidade. O paciente alegou que a lesão estava presente há mais de 2 anos, negando ser usuário de tabaco, álcool ou outras drogas; negou também trauma local associado. As histórias médica e familiar pregressa não eram contributivas. A hipótese de diagnóstico de carcinoma epidermoide foi levantada mediante as características de borda endurecida, ulceração focal e localização anatômica; contudo, o histórico de 2 anos do aparecimento da lesão se contrapunha a essa hipótese.

**Figura 1:** Aspecto clínico da lesão. (A) Visão do dorso da língua, observando alteração de volume e superfície abaulada em borda lateral direita; (B) Visão lateral direita da língua, observando área de ulceração no centro da lesão.

Foi realizada biópsia excisional sob anestesia local e em condições adequadas (Figura 2A). O fragmento medindo 0,3 x 0,3 x 0,3 cm (Figura 2B) foi devidamente acondicionado em formol 10% e enviado para análise histopatológica. Realizou-se a sutura da língua sem intercorrências (Figura 2C) e manteve-se o paciente em proservação, não havendo recidiva da lesão.

**Figura 2:** Biópsia excisional da lesão. (A) Loja cirúrgica após a remoção da lesão; (B) Lesão removida; (C) Sutura do local.

Os cortes histológicos corados em hematoxilina e eosina revelaram fragmentos de mucosa oral revestidos por epitélio pavimentoso estratificado paraceratinizado exibindo extensas áreas de acantose, hiperplasia e degeneração hidrópica (Figuras 3A e 3B). Em região subepitelial, evidenciou-se a presença de grandes vasos linfáticos dilatados e tortuosos (Figuras 3C III e 3D II), alguns deles apresentando em sua luz material eosinofílico sugestivo de linfa (Figura 3C II), bem como vasos sanguíneos de tamanhos variados, alguns se apresentando congestos, com predomínio de vasos do tipo vênulas (Figuras 3C I e 3D I) e, por vezes, se apresentavam dissociando as fibras do tecido muscular estriado esquelético presentes no fragmento tecidual. Completou o quadro histopatológico examinado a presença de tecido adiposo e tecido muscular estriado esquelético. O diagnóstico histopatológico foi de malformação venolinfática.

**Figura 3:** Fotomicrografias da lesão. (A) 2000 μm; e (B) 1000 μm. Cortes histológicos corados em hematoxilina e eosina revelaram fragmentos de mucosa oral revestidos por epitélio pavimentoso estratificado paraceratinizado exibindo extensas áreas de acantose, hiperplasia e degeneração hidrópica; CI) 200 μm. Vasos sanguíneos de tamanhos variados, alguns se apresentando congestos; CII) 200 μm. Vasos linfáticos apresentando em sua luz material eosinofílico sugestivo de linfa; CIII) 200 μm. Grandes vasos linfáticos dilatados e tortuosos; DI) 200 μm. Vasos sanguíneos de tamanhos variados, alguns se apresentando congestos; DII) 200 μm. Grandes vasos linfáticos dilatados e tortuosos.

## DISCUSSÃO

As MV são anomalias de fisiopatologia ainda imprecisa e ocorrência incomum, apresentando-se desde o nascimento, embora possam não ser aparentes e persistir quiescentes ao longo da vida adulta^6^.

Em um estudo composto por 441 pacientes, que observou malformações linfática, viu-se que 234 eram mulheres (53,1%) e 207 eram homens (46,9%), com as lesões sendo notadas com uma idade média de 1,9 anos, tendo intervalos de 0 a 28 anos, e que a região de cabeça e pescoço foi a mais acometida pelas malformações linfáticas (61,2%, n = 270), seguida pelas extremidades (17,5%, n = 77), tronco (16,1%, n = 71) ou múltiplos locais (5,2%, n = 23)^16^.

Já em um outro estudo composto por 614 pacientes, que observou malformações venosas, viu-se que 374 eram do sexo feminino (60,9%) e 240 eram do sexo masculino (39,1%). As lesões foram notadas com uma idade média de 3,1 anos, com intervalo de 0 a 46 anos. Malformações venosas envolveram as extremidades (50,3%, n = 309), cabeça e pescoço (32,7%, n = 201), tronco (9,8%, n = 60) ou múltiplos locais (7,2%, n = 44)^17^.

No tocante às MV mistas, elas são raras, principalmente em se tratando do sistema estomatognático, tendo-se ciência de apenas 11 casos relatados até o presente momento, constituindo-se de dois em corpo de mandíbula^10^^,^ 18, um em parótida esquerda^19^, um em gengiva^11^, três em mucosa bucal esquerda^3^^,^ 6 ^,^ 7, um em região sublingual^20^ e três em língua^5^^,^ 13 ^,^ 21.

Os relatos supracitados apresentam uma ampla variação de sexo, idade, tempo de evolução e localização. Tendo como achados clínicos em comum entre si e em consonância com o caso aqui exposto as lesões expansivas de superfície irregular e com alteração de cor. Já o diagnóstico clínico, em sua maioria, foi o de tumores vasculares, mas havendo, também, apontamentos para carcinoma (como o ocorrido no presente relato de caso), granuloma telangiectásico e cisto. Todos os casos mencionados no parágrafo anterior, assim como no aqui relatado, tiveram como conduta a remoção cirúrgica sem menções de recidivas; contudo, vale ressaltar que há a possibilidade de tratamentos múltiplos para malformações linfáticas, venolinfáticas ou linfático-venosas devido à recorrência.

No que tange a malformações mistas com componentes linfáticos e sanguíneos, elas geralmente são diagnosticadas no nascimento ou durante os dois primeiros anos de vida, com maior risco de se desenvolver em prematuros, embora haja evidências de casos com apresentação em idade adulta, aparentemente secundária ao trauma^9^^,^ 12 ^,^ 14.

Peculiarmente, no presente caso, apesar de ter sido notada já na fase adulta do paciente, ele negou a existência de trauma. Entretanto, além de o local ser propício a trauma, é pouco provável que uma MV que estava até então quiescente inicie alguma mudança comportamental sem que tenha havido injúria, infecção, hemorragia local ou mudanças hormonais sistêmicas, sendo possível associar a inexistência de trauma no histórico do paciente a um lapso de memória, mediante o tempo decorrido entre o aparecimento da lesão e a procura pelo serviço de saúde.

Quanto aos locais de apresentação, a ocorrência mais comum é no pescoço, também sendo relatada no duodeno, cavidade oral e região maxilofacial, cólon, bexiga, testículo e coluna vertebral^9^. Todavia, é raro o aparecimento de lesões desse porte em cavidade oral, principalmente na língua, em que, dos três casos de MV encontrados na literatura, um se trata de um caso de malformação linfático-venosa^13^ e outro está submetido a processo de recolhimento devido a publicação duplicada^5^. Torna-se, dessa forma, importante a documentação de casos clínicos como este, já que, por ser um achado incomum, há a possibilidade de confusão no momento de se levantar a hipótese de diagnóstico.

Clinicamente, o início pode variar entre uma alteração de crescimento lento ao longo de anos a um tumor de crescimento agressivo, mas sem características malignas. O tamanho desses tumores varia devido à diferente localização anatômica e associação com os tecidos vizinhos. As complicações mais comuns são hemorragia aleatória ou traumática, ruptura e infecção^15^.

Os motivos pelo quais as MV continuam a crescer são variados, podendo ser em decorrência, por exemplo, de trauma local, trombose, ressecções parciais e estímulos hormonais, acreditando-se que essas lesões aumentam de tamanho por hipertrofia em vez de proliferação hiperplásica. Clinicamente pode ser difícil prever o quanto crescerá, mas algumas lesões parecem expandir de forma invasiva em tecidos do entorno, podendo inclusive desenvolver lesões multifocais^8^.

Quanto ao tratamento, apesar das MV poderem parecer semelhantes a hemangiomas, seus cursos terapêuticos diferem^6^. As estratégias de diagnóstico e tratamento para MV devem-se basear, também, em suas características de fluxo, sendo as MV subdivididas em lesões de fluxo lento e fluxo rápido, com base na velocidade de movimento do fluido através do seu sistema capilar. Os de apresentação venosa e linfática são consideradas malformações de fluxo lento, enquanto as de apresentação arteriovenosa possuem características de fluxo rápido^8^^,^ 14 ^,^ 21. No presente caso, teve-se uma lesão de fluxo lento.

Aconselha-se o uso de exames de imagem pré-operatórios, incluindo angiotomografia, ultrassonografia e angiorressonância magnética, a fim de contribuir com o diagnóstico e planejamento da estratégia cirúrgica^14^^,^ 15 ^,^ 21. A remoção cirúrgica é o tratamento apontado como o de maior eficiência; contudo, para prevenir a recorrência, é necessária uma ressecção com margem de segurança.

## CONCLUSÃO

A complexidade da fisiopatologia das MV requer do profissional conhecimento que possibilite adequados diagnóstico e conduta terapêutica. Os achados clínicos frequentes são lesões expansivas de superfície irregular, alteração de cor, podendo ser associado a traumas e de crescimento lento, mas com a possibilidade de invadir espaços circunvizinhos, sendo raro o aparecimento de malformação venolinfática em língua, como foi o diagnóstico do caso aqui apresentado. O tratamento de escolha para o presente caso foi a remoção cirúrgica, sendo a conduta de eleição para a maioria dos casos relatados na literatura, com ausência de recidivas.
